# HIV-1 Clade B *pol* Evolution following Primary Infection

**DOI:** 10.1371/journal.pone.0068188

**Published:** 2013-06-28

**Authors:** George K. Hightower, Susanne J. May, Josué Pérez-Santiago, Mary E. Pacold, Gabriel A. Wagner, Susan J. Little, Douglas D. Richman, Sanjay R. Mehta, Davey M. Smith, Sergei L. Kosakovsky Pond

**Affiliations:** 1 Department of Medicine, University of California San Diego, La Jolla, California United States of America; 2 Department of Biostatistics, University of Washington, Seattle, Washington United States of America; 3 Life Technologies, San Francisco, California United States of America; 4 Veterans Administration San Diego Healthcare System, San Diego, California, United States of America; South Texas Veterans Health Care System and University Health Science Center San Antonio, United States of America

## Abstract

**Objective:**

Characterize intra-individual HIV-1 subtype B *pol* evolution in antiretroviral naive individuals.

**Design:**

Longitudinal cohort study of individuals enrolled during primary infection.

**Methods:**

Eligible individuals were antiretroviral naïve participants enrolled in the cohort from December 1997-December 2005 and having at least two blood samples available with the first one collected within a year of their estimated date of infection. Population-based *pol* sequences were generated from collected blood samples and analyzed for genetic divergence over time in respect to dual infection status, HLA, CD4 count and viral load.

**Results:**

93 participants were observed for a median of 1.8 years (Mean = 2.2 years, SD = 1.9 years). All participants classified as mono-infected had less than 0.7% divergence between any two of their *pol* sequences using the Tamura-Nei model (TN93), while individuals with dual infection had up to 7.0% divergence. The global substitution rates (substitutions/nucleotide/year) for mono and dually infected individuals were significantly different (p<0.001); however, substitution rates were not associated with HLA haplotype, CD4 or viral load.

**Conclusions:**

Even after a maximum of almost 9 years of follow-up, all mono-infected participants had less than 1% divergence between baseline and longitudinal sequences, while participants with dual infection had 10 times greater divergence. These data support the use of HIV-1 *pol* sequence data to evaluate transmission events, networks and HIV-1 dual infection.

## Introduction

HIV-1 is one of the fastest evolving organisms [1], [2], [3] and within a newly infected individual, quickly evolves into a highly diverse viral population [4], [5], [6]. This rapid diversification is driven by a low fidelity reverse transcriptase (2.5–3.4×10^−5^ mutations per site per generation), high replication rate (10^10^ virions produced daily), high recombination rate, genetic plasticity of viral proteins [7], and strong host immune selective pressures [3]. The proportions of sites that undergo substitutions differ both within and between HIV-1 coding regions and so studying how specific coding regions evolve provides important insight into HIV-1 pathogenesis and disease because [6], [8], [9].

Although, HIV-1 genotyping is part of the standard care in the US, its clinical use is largely limited to *pol* because of the time and cost involved in sequencing the entire HIV-1 genome. With its ubiquitous use in the surveillance and characterization of antiretroviral resistance, HIV-1 *pol* is likely the most sequenced gene from any organism (as of July 2012 there were over 167,000 sequences in the Stanford HIV Drug Resistance Database) [10]. Subsequently, analyses of large collections of *pol* sequences have provided important insights into HIV-1 epidemiology and geographic spread [11], [12], [13] however, such approaches have been limited, by the use of cross-sectional methodology and relatively limited clinical data. For example, HIV-1 *pol* evolution in acutely monoinfected vs. dual infected individuals has not been extensively examined. Our study used longitudinally collected blood samples and clinical data from newly infected ART naïve individuals to better determine intra-individual HIV-1 subtype B genetic variability, provide more accurate estimates of genetic divergence, as-well as characterize its relation to disease progression in mono- and dually-infected hosts.

## Methods

### Participants

Study participants were enrolled in the San Diego Primary Infection Cohort from December 11, 1997 through December 21, 2005, and some participants were followed up until as recently as January 26, 2010. Inclusion was limited to participants, who (1) were infected with HIV-1 clade B, (2) had a blood sample collected within a year of estimated date of infection (EDI), and (3) remained ART naïve through the sequencing of HIV-1 RNA extracted from at least two longitudinally collected blood samples. Available participant data included demographics, EDI, reported HIV risk behaviors, HLA haplotype, CD4 counts (flow cytometry), and blood plasma viral load (Amplicor, Roche) [14], [15]. Dual infection was determined by ultradeep sequencing (454 FLX Roche) of three HIV-1 coding regions (*env*, *gag* and reverse transcriptase), using a published bioinformatics protocol [16].

### HIV-1 *pol* Sequencing and Analysis

The ViroSeq™ HIV genotyping system was used for population-based *pol* sequencing per manufacturer instructions (Applied Biosystems, Foster City, CA). Sequencing was performed on an ABI 3100 Genetic Analyzer, and sequences were manually reviewed and edited (Viroseq version 2.4.2). Drug resistance was interpreted by the algorithm available with the ViroSeq™ program. Mixed bases were determined by both *basecaller* program in the Viroseq™ package, and through manual interrogation of sequence electropherograms. Sequences were aligned using a codon-based algorithm described previously [17]. HIV-1 subtype was determined using SCUEAL [18].

### Characterizing Intra-individual HIV-1 *pol* Evolution

For each participant we characterized the evolution of population HIV-1 *pol* sequences using three different approaches.

1. Phylogenetic analysis was performed using the Tamura-Nei (TN93) model [19]. This model was chosen for three reasons. First, it corrects for the primary biases of nucleotide sequence evolution in HIV-1: unequal base composition and differences in transition/transversion rates. Second, pairwise distances under this model can be computed very quickly using a closed-form solution (no numerical optimization is needed), which is why this model found wide adoption in molecular evolution studies [11], [12], [13]. Third, and most importantly, because most distances in this study are small, the impact of the substitution model on the inference is negligible (see Results for a sensitivity analysis).

- All sequences from a given patient were arranged chronologically, and the probability of evolving from the oldest to the newest sequence (given all the intermediates, if present) was estimated using maximum likelihood, using the HyPhy package [20]. Branches between any two sequences were assigned lengths based on the difference between sampling dates. For each individual **i,** we determined the nucleotide substitution rate, **r_i_** assuming strict molecular clock, i.e. that the evolutionary rate remained constant through time. We also evaluated each patient with N≥3 or more sequences for deviation from the molecular clock, by allowing each branch in the tree to have its own rate. Significance was assessed using the likelihood ratio test (LRT) with N-2 degrees of freedom [21].

1. Phylogenetic analysis was also performed using the codon-based Muse Gaut 94 model [22], which allowed us to estimate the synonymous substitution rate **s_i_** and the dN/dS ratio for each individual.
2. To draw a direct parallel with molecular epidemiological studies [11], [12], [13], which used pairwise TN93 distances to compare HIV-1 *pol* isolates, we also computed measures of overall genetic divergence for each individual. To that end, we determined the TN93 distances between the most recent and the baseline isolates and normalized it by the duration of follow-up (per year).

We took particular care to characterize genetic variability at sites with ambiguous nucleotides, as these are common in bulk HIV-1 *pol* sequences and potentially informative of population level viral polymorphism. However, standard phylogenetic approaches treat ambiguous nucleotides as partially missing data, which is conceptually equivalent to resolving ambiguities in a way to maximize sequence similarity [23]. When computing pairwise TN93 distances, we considered two alternatives. First, we resolved ambiguities to minimize pairwise differences between two sequences (*TN93-resolved*). Second, we averaged all possible complete resolutions of sequences assuming that they are equally likely (*TN93-averaged*). For instance, when comparing two sequences with ambiguous bases from the electropherogram- ACY and ARC, there are ½ (C:A) and ½ (C:G) differences between them with the resolved approach, and ½ (C:A), ½ (C:G) and ½ (C:T) differences using the averaged approach. *TN93-averaged* is necessarily greater or equal to *TN93-resolved* for any pair of sequences.

To determine the g*lobal rate of intra-individual HIV-1* pol *divergence,* we used a maximum likelihood approach and inferred a global rate of nucleotide substitutions under molecular clock with the TN93 model from all sequence data jointly (see approach (1) above). We also considered a model where the global rate differed between mono- and dually-infected individuals, and tested for equality between these rates using LRT [21].

### Intra-individual HIV-1 *pol* Evolution and Disease

HLA haplotype, HIV-1 viral load, and CD4 counts were determined as outlined above. Maximum HIV-1 viral load and minimum CD4 counts within the first year following the estimated date of infection were used as covariates. Chi-square and correlation analyses were performed to test whether HLA haplotype, maximum viral load, and minimum CD4 counts were associated with the final inferred HIV-1 substitution rates.

### The Effect of CTL-mediated Immunity on *pol* Evolution

We used the current annotation of CTL-restricted epitopes available from the Los Alamos Immunology Database (http://www.hiv.lanl.gov/content/immunology/index.html), and participant specific HLA haplotypes (two-digit resolution) to partition *pol* sequences for each individual into putative CTL-targeted epitopes (CTL^+^) and non-targeted (CTL^−^) regions. Phylogenetic rate estimation was performed, as previously described above, on CTL^+^ and CTL^−^ regions of the alignment separately, for each individual and then jointly to estimate the global substitution rate Lastly, we measured whether the mean selective pressure, estimated by the dN/dS ratio under the MG94 model, were significantly higher in CTL^+^ regions, both within individuals and globally [24].

### Subtype B Divergence and Diversity in the U.S

All unique HIV-1 *pol* sequences annotated as subtype B from the United States in the Los Alamos National Laboratory (LANL) database were downloaded [http://www.hiv.lanl.gov/]. The SCUEAL method was used as an additional filter to remove potential mislabeled or intra-subtype recombinant sequences [18]. Sequences were also removed if phylogenetic analysis using LANL *Treemaker* program demonstrated they clustered with isolates from outside the United States. [http://www.hiv.lanl.gov/components/sequence/HIV/treemaker/treemaker.html]. Finally, we computed pairwise genetic distances (TN93) between all remaining sequences, and used the resulting distribution to determine the 10%, 5% and 1% percentiles of inter-host pairwise genetic distances for subtype B HIV-1.

## Results

### Participant Characteristics

Between December 1997 and December 2005, 108 ART naive individuals were enrolled in the San Diego primary infection cohort. 15 of the 108 cohort participants were excluded from this study: nine started ART before at least one follow-up sample was taken, three were infected with non-clade B HIV-1, and for three individuals the time between EDI and first available sequence data exceeded one year (GenBank accession numbers KC814216–KC814576). The 93 eligible individuals were all male, with median viral load of 38,350 HIV RNA copies/ml (min = 50, max = 5.8×10^7^) and median CD4 556 cells/ml (min = 121, max = 1193). 16 (17%) had HIV-1 dual infection documented by ultra-deep sequencing [16], 81% were white, and 97% reported sex with other men as their major HIV risk factor (Table 1). At baseline, the median EDI was 81 days (79 for mono-infected and 94 for dually infected), and participants were observed for a median of 1.8 years (mean 2.2, SD = 1.9, min = 41 days, max = 8.6 years) following enrollment. At least one major antiretroviral resistance mutation was found in 24% of participants at enrollment, consistent with previous reports from this cohort [14], [25].

**Table 1:** Participant demographics and major outcomes.

	Singly infected	Dually infected		All	
Male gender, n (%)	77 (100%)	16 (100%)		93 (100%)	
Age, mean (SD, min, max)	33 (8.7, 19, 58)	34 (8.2, 22, 54)		33 (8.6, 19, 58)	
Risk, Sex with Men, n (%)	74 (96%)	16 (100%)		90 (97%)	
Race, white, n (%)	61 (79%)	14 (88%)		75 (81%)	
BL CD4, median (min, max)	552 (121, 1180)	571 (263, 1193)		556 (121, 1193)	
BL RNA, median (min, max)	41,600 (50, 58×10^6^)	34,900 (779, 21×10^6^)		38,350 (50, 58×10^6)^	
Any resistance, n (%)	19 (25%)	3 (19%)		22 (24%)	
EDI in days to first visit Median (min, max)	79 (11, 189)	94 (11, 177)		81 (11, 189)	
EDI in days to last follow-up Median (min, max)	741 (116, 3274)	617 (236, 2351)		696 (116, 3274)	
TN93 phylogenetic distance. Substitutions/site/year. Mean (Median; 5%–95%)	0.0007 (0.0004; 0.0000–0.0031)	0.0192 (0.0014; 0.0000–0.0905)	0.0039 (0.0004; 0.0000–0.0052)		
MG94 phylogenetic distance. Synonymoussubstitutions/site/year. Mean (Median; 5%–95%)	0.0003 (0.0000; 0.0000–0.0018)	0.0131 (0.0003; 0.0000–0.0603)	0.0025 (0.0000; 0.0000–0.0036)		
TN93-resolved (first, last). Substitutions/site/year.Mean (Median; 5%–95%)	0.0006 (0.0003; -0.0000–0.0031)	0.0103 (0.0005; -0.0000–0.0726)	0.0023 (0.0003; -0.0000–0.0033)		
TN93-averaged (first, last). Substitutions/site/year.Mean (Median; 5%–95%)	0.0025 (0.0014; 0.0003–0.0062)	0.0154 (0.0026; 0.0008–0.0774 )	0.0047 (0.0015; 0.0003–0.0120)		

### Intra-individual HIV-1 *pol* Genetic Evolution

**Figure 1:**
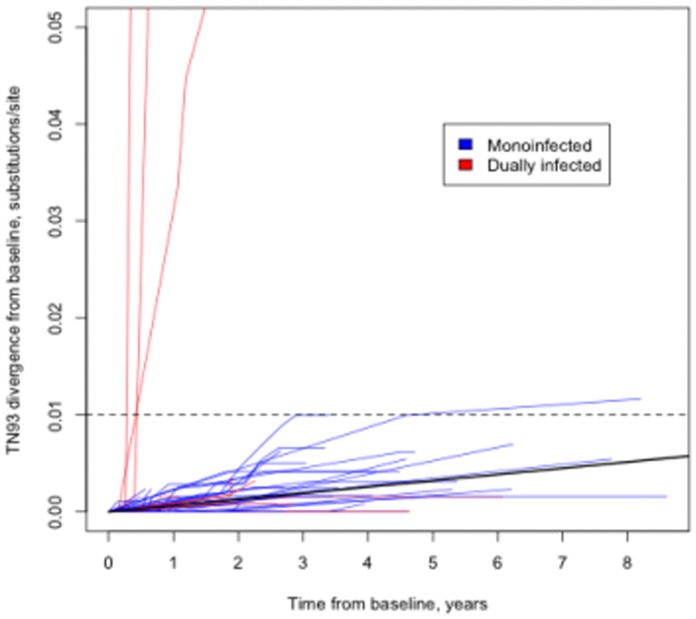
Divergence from baseline, based on the phylogenetic estimates under the TN93 model without assuming a molecular clock for each of the 93 study participants. The solid line represents the mean evolutionary trajectory inferred from mono-infected samples. The dashed horizontal line depicts the 1% genetic distance cutoff, used previously to infer potential epidemiological linkage [11]–[13].

HIV-1 *pol* population-level evolution occurred at a low rate, with 37/93 individuals showing no nucleotide substitutions (under the phylogenetic TN93 model) over the entire period of observation. The median substitution rate (including mono and dual-infected individuals) was less than 0.01 substitutions/site/year for all four genetic distance estimates (Table 1). The estimated global substitution rate for mono-infected individuals was 7.2×10^−4^ (5%–95% = 6.1×10^−4^–8.4×10^−4^) substitutions/site/year, which is slower than the estimates of the substitution rate based on comparing pol isolates from different individuals under relaxed molecular clock models (1-2×10^−3^, e.g. [11], [26], [27]). The overall rate for dually infected individuals was 9.7×10^−3^ (8.7×10^−3^–1.1×10^−2^) (Figure 1). Global substitution rates for mono and dually infected individuals were significantly different (p<0.001, LRT). It is important to note that for dually infected participants this rate estimate is confounded by the introduction of the second divergent strain, and does not suggest that the intrinsic substitution rate is an order of magnitude faster in those patients. In many of the participants studied, nucleotide substitutions did not occur at a constant rate–in individuals with ≥3 timepoints available, the hypothesis of a strict molecular clock could be rejected in 18 of the 39 mono-infected (at p≤0.05) and 5 of 9 dually infected cases. As expected, on average, *pol* evolved under purifying selection and we could not reject the hypothesis of purifying selection or neutral evolution for any of the within-host samples (p≤0.05, LRT).

### Effect of Evolutionary Model

Since a poorly selected model of evolution can lead to statistical issues during inference [11]–[12], we assessed the sensitivity of within-host rate inference in the phylogenetic context to the choice of evolutionary model. We compared TN93 to both simpler (JC69 and F81) and more complex (GTR, GTR+G) models, and found that the inference was essentially unaffected. The estimates of within-host substitution rates under all 4 models were nearly perfectly linearly correlated (adjusted R^2^ of 0.98 or greater), and the only substantial differences were observed for 2 dually infected patients with ≥3% annual rate (between GTR+G and all other models, see Figure S1). This is not surprising, since similar model performance is expected in the setting of very low sequence divergence. Thus, we conclude that the choice of the TN93 model does not unduly influence our results.

### Effect of Distance Metric

Evolutionary rate estimates from the phylogenetic TN93 and *TN93-resolved* distances were strongly correlated. This was especially true when restricting the analysis to the 0–0.02 range, which included the vast majority (89/93) of individuals (p<0.001, adjusted R^2^ = 0.89, linear model). Similarly, when restricted to the 0–0.02 range, estimates from phylogenetic TN93 correlated with phylogenetic MG94 (synonymous rates): p<0.001, adjusted R^2^ = 0.73). Under the *TN93-averaged* metric, non-zero distances were assigned to all but 2 individuals and so linear relationships to the other metrics were poor; however, rank-based testing provided clear evidence of correlation with the other metrics (p<10^−9^ in all pairwise comparisons, Wilcoxon signed-rank test).

### The Impact of CTL-mediated Immune Selection on HIV-1 *pol* Evolution

We found that the rates of evolution within *pol* regions containing epitopes that have been reported as targeted by the individual-specific HLA alleles (2 digit resolution) were consistently higher than outside those regions. Globally, the rate of nucleotide evolution for monoinfected individuals was 9.5×10^−4^ (7.5×10^−4^–1.2×10^−3^) substitutions/site/year in HLA regions, and 6.0×10^−4^ (4.8×10^−4^–7.3×10^−4^). For dually infected individuals, the estimates were 1.1×10^−3^ (9.5×10^−4^–1.3×10^−3^), and 9.0×10^−4^ (7.8×10^−4^–1.0×10^−3^), respectively. In both cases, the rates were significantly higher in CTL^+^ regions based on the phylogenetic likelihood ratio test, p = 0.005 (monoinfected), p = 0.04 (dually infected). A similar comparison within individuals revealed that CTL^+^ region rates were faster in 14 cases and slower in 3 cases (p≤0.05, LRT).

Consistent with multiple literature reports (i.e. [25], [28], [29], [30], [31]), we found that selective pressure was elevated in CTL^+^ regions. Joint estimation for monoinfected individuals, which allowed synonymous substitution rates to vary among individuals, yielded dN/dS = 0.98 (0.73–1.28) for CTL^+^ and dN/dS = 0.49 (0.37–0.63) for CTL^−^ (the hypothesis that these values were the same can be rejected at p<0.001 using LRT). For dually infected individuals, these values were 0.15 (0.12–0.18), and 0.31 (0.24–0.40), also significantly different (p<0.001). dN/dS was also markedly (p≤0.05, LRT) elevated in CTL^+^ regions for 11 individuals.

### Intra-individual HIV-1 *pol* Evolution and Disease Progression

Genetic divergence and nucleotide substitution rates (*TN93-resolved*, *TN93-averaged*, MG94) tended to be smaller for individuals with HLA haplotype B27 and B51 (p-values between 0.05 and 0.10) when all individuals were considered. It is important to note, this finding was likely driven by a few dually infected individuals with high substitution rate. When only mono-infected individuals were considered (all p-values>0.20) in similar analysis the results were not statistically significant. In mono-infected individuals *TN93-averaged* substitution rates were inversely and significantly associated with the observed nadir CD4 count within one year of EDI (ρ = -0.37, p<0.001). There was no significant correlation between nadir CD4 count and nucleotide substitution rates for dually-infected individuals or for all individuals considered together. Substitution rates were not associated (all p>0.1) with CD4 count, viral load, or the presence of transmitted resistance associated mutations (data not shown).

### U.S. HIV-1 Subtype B Divergence and Diversity

The analysis of the distribution of genetic distances between sequence from curated set of HIV-1 subtype B *pol* isolates collected in the United States from LANL database, placed the estimates of the 10%, 5% and 1% percentile at 0.044, 0.040 and 0.033, respectively. In other words, the genetic distance between 99% of any two random HIV-1 *pol* sequences from U.S. individuals with subtype B infection is expected to be 3.3% or greater, which exceeds the intra-host viral genetic divergence observed in all of the mono-infected patients in this study.

### Conclusions

The utility and widespread use of HIV-1 *pol* sequencing has facilitated the study of HIV-1 molecular epidemiology and evolution [11], [12], [13]; however many of these studies have relied on non-longitudinal and limited clinical data. To further characterize intra-host HIV-1 clade B *pol* evolution in recently infected individuals, who were previously characterized as mono- or dually- infected, we analyzed *pol* sequences generated from longitudinally collected blood samples in conjunction with HLA haplotype and markers of disease progression.

Overall, HIV-1 *pol* evolution was slow, with the gene accumulating fewer than 0.01 substitutions/nucleotide/year. Estimated global substitution rate for mono-infected individuals was 7.2×10^−4^ (5%–95% = 6.1×10^−4^–8.4×10^−4^) substitutions/site/year, which is slower than previous estimates that relied on comparing *pol* isolates from different individuals under relaxed molecular clock models (1-2×10^−3^, [11], [26], [27]). For the majority (52%) of mono-infected participants, population-level *pol* sequences were unchanged after resolving ambiguous nucleotides. Even after an average of 19 months of observation and nearly 9 years of follow-up, divergence remained below 2% for all mono-infected individuals. These findings indicate that the 1% genetic distance cutoff invoked in previous epidemiological linkage studies is likely a conservative estimate to infer individuals belonging to the same transmission network [32], [33], [34].

In line with previous studies, we observed insignificantly faster nucleotide substitution and higher dN/dS rates within putative CTL targeted regions of *pol,* indicating that immune selective pressure is an important factor driving divergence [25], [29], [30], [31]. However, for mono-infected individuals *pol* substitution rates were associated with nadir CD4 count, but not with HLA haplotype, viral load or the presence of transmitted resistance associated mutations. While, the observed relationship between higher substitution rates and nadir CD4, likely reflects waning CTL mediated selection pressure, as others have reported [30]–[32], it is difficult to interpret the relationship between *pol* evolution and other markers of disease progression given there was little or no change in observed viral genotype for most individuals studied. The low observed viral change is an important limitation of this observation and should be pursued in future investigations.

HIV dual infection is defined by the presence of two (or more) concurrently circulating and genetically distinct viral populations in an individual [13], thus it is not surprising that this study inferred a higher rate of viral genetic divergence for individuals with dual infection. The pronounced differences in genetic divergence among dual infected individuals, likely reflect instances in which the original HIV-1 *pol* sequence has been replaced with the superinfecting strain [16]. Of note, the four dually infected individuals with highest genetic divergence did not differ significantly from the other 12 cases with respect to disease progression (data not shown), which is different from previous reports [16], but this is likely secondary to sample size. Significantly higher dN/dS substitutions within CTL epitopes compared to non-CTL regions, provided clear evidence of host specific selection contributing to divergence, which mirrored our findings in mono-infected individuals.

In summary, this study provides additional evidence that dual infection provides increased amounts of viral genetic material to the infecting viral population and allows for faster rates of observed molecular evolution. For mono-infected individuals, however, this study provides important empirical evidence that the rates of intra-host *pol* evolution are slow, and lends credence to using *pol* sequence similarity for transmission network or linkage inferences, even after long periods between sampling and putative transmission events.

## Supporting Information

Figure S1 **Comparison between nucleotide models demonstrate that inference is insensitive to the choice of substitution model.** This is mostly because for very low divergence within individuals, even the simplest models (e.g. F81) provide an adequate approximation to the most general (GTR+G) model.
(TIF)
