# Supplementary figures and images for: HIV-1 Clade B pol Evolution following Primary Infection
Source: PLoS One. 2013 Jun 28;8(6):e68188. doi: 10.1371/journal.pone.0068188 (PMC3695957; doi:10.1371/journal.pone.0068188)

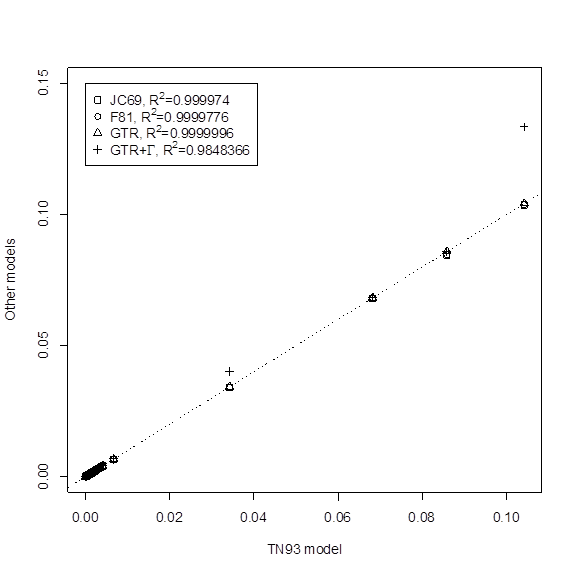

Supplement: Figure S1 — Comparison between nucleotide models demonstrate that inference is insensitive to the choice of substitution model. This is mostly because for very low divergence within individuals, even the simplest models (e.g. F81) provide an adequate approximation to the most general (GTR+G) model. (TIF) [file pone.0068188.s001.tif]
